# Long-term Sonographic and Serological Follow-up of Inactive Echinococcal Cysts of the Liver: Hints for a “Watch-and-Wait” Approach

**DOI:** 10.1371/journal.pntd.0003057

**Published:** 2014-08-14

**Authors:** Luca Piccoli, Francesca Tamarozzi, Federico Cattaneo, Mara Mariconti, Carlo Filice, Antonella Bruno, Enrico Brunetti

**Affiliations:** 1 Department of Infectious Diseases, IRCCS San Matteo Hospital Foundation, Pavia, Italy; 2 WHO Collaborating Centre for Clinical Management of Cystic Echinococcosis, Pavia, Italy; 3 Department of Clinical, Surgical, Diagnostic and Pediatric Sciences, University of Pavia, Pavia, Italy; 4 Laboratory of Parasitology, IRCCS San Matteo Hospital Foundation, Pavia, Italy; Lindsley F. Kimball Research Institute, New York Blood Center, United States of America

## Abstract

Human cystic echinococcosis is a chronic, complex and neglected infection. Its clinical management has evolved over decades without adequate evaluation of efficacy. Recent expert opinion recommends that uncomplicated inactive cysts of the liver should be left untreated and solely monitored over time (“watch-and-wait” approach). However, clinical data supporting this approach are still scant and published mostly as conference proceedings. In this study, we report our experience with long-term sonographic and serological follow-up of inactive cysts of the liver. From March 1994 to October 2013, 38 patients with 47 liver cysts, diagnosed as inactive without any previous treatment history, were followed with ultrasound and serology at 6–12 months intervals for a period of at least 24 months (median follow-up 51.95 months) in our outpatient clinic. In 97.4% of patients, the cysts remained inactive over time and in only one case was reactivation of the cyst detected. No complications occurred during the time of monitoring. During follow-up, serology tests for CE were negative at diagnosis or became negative in 74.1% and were positive or became positive in 25.9% of cases. Patients with inactive cysts on ultrasound but positive serological tests were also investigated by CT scan (chest and abdomen) to rule out extra-hepatic cyst localization. This study confirms the importance of a stage-specific approach to the management of cystic echinococcosis and supports the use of a monitoring-only approach to inactive, uncomplicated cysts of the liver. It also confirms that serology plays only an ancillary role in the clinical management of these patients, compared to ultrasound and other imaging techniques. The implications of these findings for clinical management and natural history of cystic echinococcosis are discussed.

## Author Summary

Human cystic echinococcosis (CE) is a chronic, complex and neglected parasitic infection presenting mostly as hepatic cysts, which are staged by ultrasound. Recent expert opinion recommends that uncomplicated inactive cysts should be left untreated and solely monitored over time, using the so-called “watch-and-wait” approach. Currently, no reliable biological marker of cyst activity is available. Positive antibody titers may persist for years even after removal of a cyst; therefore a long-term follow-up is required to assess the evolution of the cyst's biological activity over time. The watch-and-wait approach to inactive hepatic CE cysts is increasingly used in selected cases in referral centers; however, no data on its safety and effectiveness has yet been published. We retrospectively studied 47 inactive uncomplicated cysts at diagnosis, managed by a watch-and-wait approach, with a median follow-up of 51.95 months. We observed that these cysts remained inactive over time in almost all cases, without any complications. These results support the watch-and-wait approach to these cysts. Furthermore, we confirmed that serology only plays an ancillary role in the clinical management of these patients, compared to ultrasound and other imaging techniques.

## Introduction

Cystic echinococcosis (CE) is a chronic, complex and neglected infection caused by *Echinococcus granulosus*, a cestode with a worldwide distribution affecting an estimated 1.2 million people, mainly in pastoral communities [1], [2], [3]. Its life cycle develops between the dog and other canids, which harbor the adult tapeworm in the intestine and shed parasite eggs in feces, and herbivores that are intermediate hosts. Humans are dead-end occasional intermediate hosts and acquire the infection through accidental ingestion of *Echinococcus* eggs. In humans, the larval stage of the tapeworm forms a cyst that is located in the liver in about 80% of cases but may occur in almost any organ [4]. Although often asymptomatic, this chronic infection accounts for an estimated 3.6 million DALYs (Disability Adjusted Life Years) lost globally every year [2].

**Figure 1:**
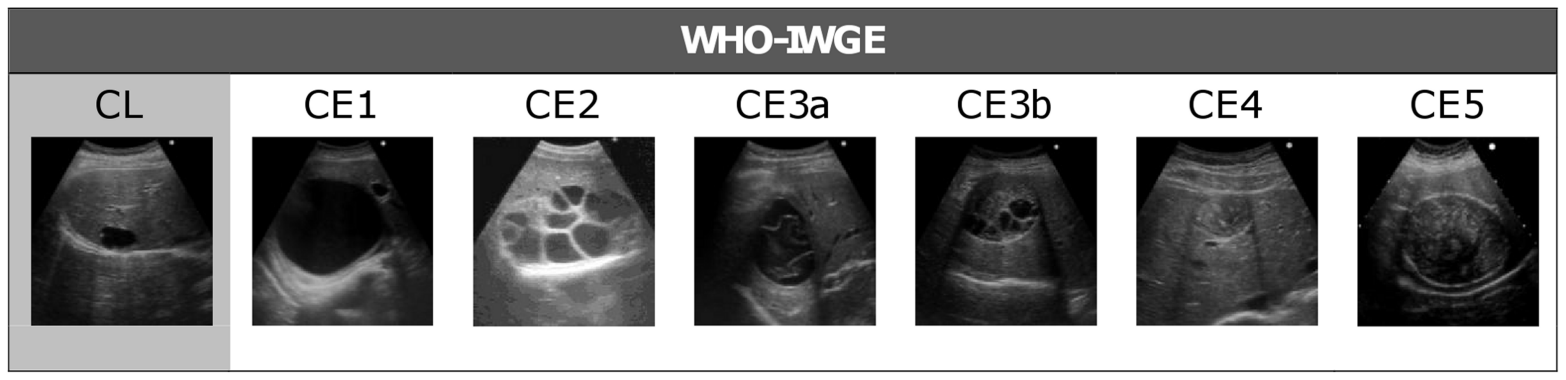
WHO-IWGE ultrasound classification of echinococcal cysts. CE1 and CE2 (active cysts), CE3A and CE3B (transitional cysts), and CE4 and CE5 (inactive cysts) [8].

Diagnosis and clinical management of hepatic CE currently rely on imaging techniques, especially ultrasound (US) [5], and a number of sonographic classifications of CE have been proposed in the past 30 years [6], [7], [8]. The current classification, issued by the WHO-IWGE (World Health Organization-Informal Working Group on Echinococcosis), allows the distinction into active (CE1 and CE2), transitional (CE3) and inactive (CE4 and CE5) cyst stages [8] (figure 1). This classification is supported by the different biological activity demonstrated in distinct cyst stages [9], which in turn supports the clinical observation that different stages respond differently to non-surgical therapy [10]. Altogether, these support the concept of a stage-specific approach to treatment, at least for hepatic locations [4], [10].

According to the stage-specific approach proposed by WHO-IWGE, uncomplicated cysts of the liver should be treated by non-surgical options (percutaneous drainage and medical treatment with benzimidazoles), while surgery should be used when complications are present and in other selected circumstances [4], [11]. Furthermore, recent expert opinion also recommends that inactive CE4-CE5 cysts that are asymptomatic and uncomplicated should be left untreated and solely monitored regularly by ultrasound, using the so-called “watch-and-wait” approach [4], [11], [12]. However, these different options have never been systematically evaluated and properly compared, at least in part due to the chronicity of the infection and its evolution, which would require years-long follow-up to be properly evaluated, a requirement that is extremely difficult to achieve. As a consequence, the “best” treatment for echinococcal cysts is still the subject of debate. The watch-and-wait approach for inactive hepatic CE cysts is increasingly used in selected cases in referral centers; however medical or surgical treatments are still commonly performed elsewhere for these cases.

Long-term follow-up with ultrasound is required to assess the evolution of the CE4–CE5 cyst biological activity over time. Serological tests may be useful to confirm US diagnosis of CE, but they are not reliable for assessing cyst viability and often a positive serology induces clinicians unfamiliar with CE to treat inactive cysts unnecessarily, while antibody titers may persist for years even after complete surgical removal of the cyst [13], [14]. Moreover, serological tests lack standardization and show variable diagnostic performance, which depends on many factors such as prevalence of infection, cross-reaction with other parasites, and stage, location, and size of the cysts [15], thus making their results and implications for clinical management difficult to interpret.

Although published studies based on ultrasound surveys support the use of watchful monitoring of asymptomatic inactive CE [16], [17], to date, no data on the safety and effectiveness of the watch-and-wait approach have been published from a clinical setting in clinically well-defined patients. To start filling this gap, we report our experience with long-term sonographic and serological monitoring of a well-defined group of patients with uncomplicated hepatic inactive cysts that were in the inactive stage at the time of first diagnosis.

## Methods

### Ethics statement

The study protocol was approved by the ethical committee of San Matteo Hospital Foundation, Pavia, Italy, and all patients gave their written informed consent.

### Inclusion criteria

Clinical records of patients who were diagnosed in our clinic with exclusively inactive echinococcal cysts (i.e. cysts that reached the inactive stage spontaneously at the time of diagnosis) of the liver until October 2013 were extracted from our electronic archive. Data (demographic and contact details, characteristics of the cysts at diagnosis and during follow-up, serology, treatment, and development of any symptoms or complications) have been routinely recorded for CE patients at each visit from March 1994 and were available for analysis. Our Centre has been the WHO collaborating Centre for the Clinical Management of Cystic Echinococcosis since 2009. In the last 30 years, more than 690 patients with CE have been seen and clinically followed, both from Italy (about 2/3) and other endemic countries (about 1/3), with 24 new diagnoses of CE in 2012 alone. Patients were selected among those who met the following inclusion criteria: (i) harboring exclusively uncomplicated inactive hepatic CE4 or CE5 cysts that were in the inactive stage at the time of first diagnosis, (ii) follow-up defined as abdominal ultrasound and serological tests performed every 6–12 months in our center, and (iii) minimum length of follow-up of 24 months in our center. Patients with cysts that became inactive over time, spontaneously or as a result of treatment, and/or with concomitant presence of active or transitional cysts in the liver or elsewhere, were excluded from the analysis.

### Ultrasound

All patients were examined by an infectious disease clinician with long-standing experience in US and clinical management of CE (EB) using a commercially available US scanner with 3.5–5 MHz convex probes (Hitachi 19, Hitachi, Japan and Aloka ProSound ALPHA 10, Tokyo, Japan). For each patient, number, stage, size and location of the cysts were recorded. Solid cysts were classified as CE4 or CE5 according to the WHO-IWGE standardized US classification for CE [8] (Figure 1).

### Serology

All patients were tested for anti-*Echinococcus* antibodies by commercial IgG Enzyme-Linked Immunosorbent Assay (ELISA, Cypress Diagnostic, Langdorp, Belgium, marked CE) in the parasitology laboratory of our hospital, according to manufacturer's instructions. Because cut-off values changed in 2003, here we evaluated the serology results only of those patients diagnosed after this date.

### Data analysis

For each patient, demographic details, characteristics of the cysts at diagnosis and during follow-up, previous treatments, and development of any symptoms or complications were obtained. Sonographic and serological changes, if any, during the follow-up were also recorded. Median follow-up and inter-quartile range (IQR) were calculated only from the follow-up time performed by regular visits in our center. The visualization of daughter cyst development within the cyst, i.e. a stage shift from CE4 or CE5 to CE3b, was considered a reactivation. The McNemar's test was applied to analyze the difference in cyst stage at diagnosis and last follow-up visit for each patient. Quantitative serological results were summarized as negative (constantly below cut-off for positivity), positive (constantly above cut-off), negativization or positivization (evolution from above to below cut-off values or *vice versa*, respectively, at least once during follow-up). Patients with inactive cysts but positive serological tests were investigated by CT scan (chest and abdomen) to rule out the presence of extra hepatic cysts that might have remained undiagnosed, which could explain serological positivity.

Patients included in the study series were divided into three groups, as follows: i) patients still in follow-up in our centre in October 2013, ii) patients no longer in regular follow-up but reached by telephone, and iii) patients not reachable by telephone. Medians between groups were compared using the Kruskal-Wallis test and the Mann-Whitney U test. Percentages between groups were compared using the Chi-square tests. Where appropriate, the Bonferroni correction was applied to adjust for multiple comparisons. Statistical analysis was carried out in SPSS Statistics 17.0 (IBM). A *p*-value≤0.05 was considered significant.

## Results

### Clinical and follow-up characteristics of included patients

**Figure 2:**
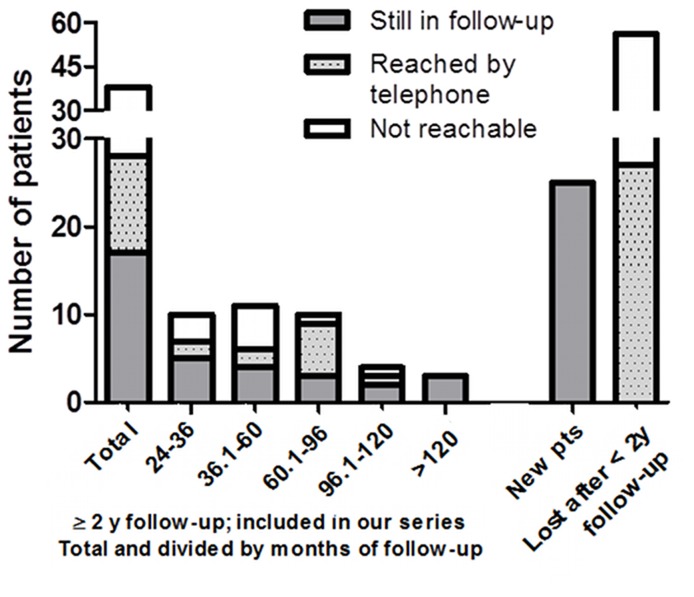
Distribution of follow-up length in our centre and loss to follow-up of patients diagnosed with inactive CE cysts during the investigated period. Patients included in our case series, are shown as total and divided by follow-up length groups. New patients group refers to patients first visited after Oct 2011. Pts = patients.

From March 1994 to October 2013, 127 patients with exclusively inactive, uncomplicated liver cysts, which were already inactive at first diagnosis, were seen in our clinic. The demographic and clinical characteristics of the 38 patients included in the analysis are summarized in table 1, while 89 did not meet the inclusion criteria (table 2). All patients were diagnosed with inactive cysts during sonographic scans performed to investigate symptoms compatible with, but ultimately unrelated to, CE (abdominal pain, fever, increase of transaminases). The median follow-up period in our center was 51.95 months (IQR 35.69–95.04 months) (table 1). The distribution of follow-up length and present follow-up situation of patients included in the study series is shown in figure 2. The 38 selected patients harbored a total of 47 hepatic cysts: 26 CE4 cysts and 21 CE5 cysts. Twenty nine patients had only one cyst, while 9 patients had two cysts (table 1). In 37 (97.4%) patients, the cysts remained inactive throughout the observation period, and no change from CE4 to CE5 was recorded. In 1 patient a cyst reactivation was detected, from inactive CE4 to transitional CE3b, after 2 years of follow-up. This patient had never been treated and the reactivated CE3b cyst has remained stable to date (7 years after reactivation) in the absence of treatment. The difference in cyst stage between diagnosis and last visit was not statistically significant.

**Table 1:** Summary of patient characteristics, US cyst appearance, and imaging and serological behaviour over time of patients included in the study series.

**PATIENTS**			
**Sex**	15 males (39.5%)		23 females (60.5%)
**Mean age at diagnosis**	50 years (range: 14–86 y)		
**Birth place**	Italy: 32 (84.2%)		Other countries:6 (15.8%)
**Place of diagnosis**	At our clinic: 27 pts (71.1%)		In other centers: 11 (28.9%)
**Still in follow-up in 10/2013**	Yes 17 (44.7%)		No 21 (55.3%)
			10 not reachable
			11 contacted by telephone
**Reasons for not attending follow-up**	3 followed in other centers (no reactivation or complication reported		
	8 asymptomatic and did not consider a visit necessary		
**Median follow-up (IQR)**			
from diagnosis	59.27 months (IQR 35.96–95.04)		
from 1^st^ control in our center	51.95 months (IQR 35.78–87.95)		
of patients still in follow-up	70.30 months (IQR 26.70–117.20) from diagnosis		
	59.20 months (IQR26.60–104.5) from 1^st^ control in our center		
of patients reached by phone	66.93 months (IQR 39.10–85.73) from diagnosis		
	66.93 months (IQR 39.10–85.73) from 1^st^ control in our center		
of patients not reachable	40.00 months (IQR 36.74–68.94) from diagnosis		
	40.45 months (IQR 36.01–51.98) from 1^st^ control in our center		
**CYSTS**			
**Number per patient**	29 pts with 1 cyst (76.3%)		9 pts with 2 cysts (23.7%)
**Mean diameter**			
all cysts	55.09 mm (range: 15.5–100 mm)		
CE4	52.14 mm (range: 15.5–91.93 mm)		
CE5	58.46 mm (range: 22–100 mm)		
**Liver location**			
all cysts	Right lobe: 36 (75%)	Left lobe: 5 (10.4%)	IV segment: 7 (14.6%)
CE4	21 (80.2%)	2 (9.5%)	3 (14.3%)
CE5	15 (68.2%)	3 (13.6%)	4 (12.2%)
**Type**			
all cysts	CE4 (solid): 26 (55.3%)		CE5 (solid and calcified): 21 (44.7%)
in patients still in follow-up	CE4: 7 (41.17%)		CE5:10 (58.83%)
in patients reached by phone	CE4: 5 (45.45%)		CE5:6 (54.55%)
in patients not reachable	CE4: 7 (70%)		CE5:3 (30%)
**Stability over time**	Stability: 37 pts (97.4%)		Reactivation: 1 pt (2.6%)
**Complications**	0 (0%)		
**SEROLOGY (n = 27)**			
	negative: 19 pts (70.4%)		positive: 6 pts (22.2%)
	negativization: 1 pts (3.7%)		positivization: 1 pts (3.7%)

Percentages refer to the number of subset observations on the total number of observations per row. Unless indicated otherwise, numbers refer to patients. Pts = patients.

**Table 2:** Summary of patient characteristics and follow-up details of all subjects diagnosed with inactive CE cysts during the investigated period.

	Included N = 38 N (%)	Excluded N = 89 N (%)	All patients N = 127 N (%)
**REASON(S) FOR EXCLUSION**			
Follow-up <24 m ended before Oct 2011 (lost to follow-up)	-	61 (68.5%)	61 (48.0%)
Follow-up <24 m started after Oct 2011 (new diagnoses)	-	28 (31.5%)	25 (22.0%)
Treatment before visit in our center	-	16 (17.9%)	16 (12.6%)
**STILL IN FOLLOW-UP IN OUR CENTER**	17 (44.7%)	28 (31.4%)	45 (35.4%)
**PHONE INTERVIEW**			
Not reachable	10 (26.3%)	30 (33.7%)	40 (31.5%)
Deceased	0	2 (2.2%)	2 (1.5%)
Follow-up in other centers	3 (7.9%)	16 (17.9%)	19 (14.9%)
Judged further visits not needed (asymptomatic)	8 (21%)	13 (14.6%)	21 (16.5%)
**REACTIVATION/COMPLICATIONS**			
Total	1 (2.6%)	1 (1.1%)	2 (1.5%)
Assessed in our center	1 (2.6%)	0	1 (0.8%)
Not assessed in our center	0	1 (1.1%)	1 (0.8%)

Results refer to patient numbers and percentages of patients within each group (included in our case series, excluded from our case series, and all patients combined, defined in columns). The sum of values within columns may exceed total number of patients per column as one patient may belong to different categories.

Twenty one (55.2%) patients who were included in our study were not seen during follow-up for >12 months after the last control. To gain a more complete understanding of the follow-up outcome, and to assess the presence of systematic differences between patients still visited in our center and those lost to follow-up, these were re-contacted by telephone. Ten patients (47.6% - 7 Italians and 3 foreigners) could not be reached at the telephone number provided at the time of the last visit, while 11 (52.4%) could be interviewed, with no complications reported (table 1). Age, sex, median follow-up length, and CE4 or CE5 cyst stage distribution were not statistically different between patients groups, while patients lost to follow-up but reached by telephone had statistically higher numbers of cysts compared to the other two groups (p = 0.03 for both comparisons).

### Follow-up characteristics of patients not included in the study series

Eighty nine patients did not meet the inclusion criteria (table 2 and figure 2). To gain a more complete understanding of the follow-up outcome, we re-contacted by telephone the 61 patients who were not seen during follow-up for >12 months from their last visit. Thirty patients (15 Italians and 15 foreigners) could not be reached at the telephone number provided at the time of the visit. The results of the interview of the remaining 31 patients who could be reached by telephone are shown in table 2. Only one of these patients, currently followed in another hospital, reported to have suffered from complications, but it was not possible to clarify the nature and relation to CE cyst by telephone.

### Serology

Serology results (table 1) were remarkably stable over time. ELISA was persistently negative in 19 patients (70.4%), positive in 6 (22.2%), while 2 (7.4%) patients converted/reverted. Of note, the OD value of the patient showing CE4 to CE3b change was negative at the time of reactivation, and has remained so. These results were confirmed by a second serology test (IHA, Cellognost Echinococcosis; Dade Behring, Newark, USA; data not shown). Patients with positive serology for CE did not harbor cysts in extra hepatic locations as shown by abdominal and thoracic CT scans.

## Discussion

Cystic echinococcosis is among the most neglected parasitic diseases, and development of new drugs and other treatment modalities for this infection receives very little attention [2]. In addition, CE is a complex disease due to the possible involvement of different organs and tissues, the presence of different cyst stages ranging from active to inactive, and its wide spectrum of clinical presentations ranging from asymptomatic to life-threatening complications such as anaphylactic shock and pulmonary embolism [4]. Moreover, CE infection and its evolution is chronic, further hampering its study, and would require many years follow-up, which is extremely difficult to achieve. As a consequence of both neglect, complexity, and chronicity of CE, clinical management procedures have evolved over decades without adequate evaluation of essential features such as efficacy, rate of adverse reactions, relapse frequency and cost.

The advent of modern imaging techniques, in particular ultrasound, represented a breakthrough in the diagnosis, treatment and follow-up of patients with CE. As a consequence, clinicians have been striving for the past 30 years for an imaging-based classification of CE cysts to harmonize the interpretation of scientific studies and to guide clinical management of CE [6], [7], [8]. Concomitantly, progress was made to correlate individual stages of these classifications with the natural history of the cyst and involution processes accelerated by treatment, although controversy still exists on this subject [18].

Surgery, percutaneous interventions and chemotherapy with benzimidazoles are three available treatment options; however they have never been systematically evaluated and properly compared, at least in part due to the chronicity of CE infection, which would require many years- if not decades of regular follow-up, very difficult to achieve in a large number of patients. Despite this, the WHO-IWGE classification, which groups cysts into active, transitional and inactive, has made a rational, stage-specific approach possible [4], [8].

Recently, experts have suggested that uncomplicated inactive cysts should be left untreated and simply monitored by ultrasound, a “watch-and-wait” approach alternative to treatment [4], [11]. As illustrated by Junghanss *et al.* [19], the idea of leaving uncomplicated, inactive cysts untreated and solely monitored over time follows the observation that a good proportion of cysts become spontaneously inactive without any treatment and such cysts are likely to remain stable over time. Although the watch-and-wait approach is being increasingly used in selected cases in referral centers, and published studies based on ultrasound surveys support the use of watchful monitoring of asymptomatic inactive CE, no data on the safety and effectiveness of this approach in well-defined patients in a clinical setting have been published.

In almost all (97.4%) patients with spontaneously inactive cysts followed in our center for at least two years over a period of 19 years, the cysts remained inactive. In only one patient were we able to detect reactivation of a single CE4 inactive cyst to a transitional CE3b type after a 2 year follow-up period. Equally important is the fact that no complications were recorded and the patients had no symptoms related to the presence of the cyst in the liver during their follow-up. Furthermore, although the administration of albendazole to eight patients for 1 to 6 months before being seen at our center excluded them from the study series, this treatment did not produce any noticeable change in the features of their already-inactive cysts.

These results certainly support the watch-and-wait approach to spontaneously inactivated CE4–CE5 cysts, notwithstanding the limitations of our retrospective study. Loss to follow-up of CE patients (64.6%), a problem commonly faced by clinicians, is not limited to patients managed with watch-and-wait in our experience. The dynamic of loss to follow-up in our center seems bimodal, as shown in figure 2, with most patients either never entering a follow-up after the first visit (54.9% of all patients lost to follow-up) or interrupting the follow-up after a few years of regular visits. About half of patients included in our series who did not have a follow-up visit in the year or more before data cut-off could be reached by telephone, and, of these, half declared that they did not seek further medical advice because they were asymptomatic and they did not consider a control visit necessary, while the other half are routinely followed in other centers. The remaining patients could not be reached because of change of contact information, and this was found equally in Italians and non-Italians. These figures apply when considering either patients included in our case series, in those excluded, or both groups combined. All but one of patients reached by telephone were declared to be asymptomatic and those followed elsewhere have their cysts unchanged. Although the information collected by telephone interview is limited, we focused on three conditions (whether follow-up was performed elsewhere; reasons for absence to follow-up; and, patient's health status as far as CE-related symptoms were concerned) which we believed could be reasonably assessed without a first-hand visit. If limited to those people included in our study series, it is possible to speculate that the same absence of complications applies to the subgroup not reachable by telephone, considering the homogeneity of baseline conditions with those patients still in follow-up or reached by telephone. Conversely, the fate of those patients lost to follow-up very shortly after the first visit cannot be extrapolated. Clearly, an improvement in the doctor-patient relationship and a better explanation of the necessity of regular follow-up of even inactive and asymptomatic CE cysts are needed. Indeed, the only cyst reactivation we observed was detected after 2 years of follow-up, highlighting the importance of a long-term follow-up of these patients, to detect promptly any reactivation and complication.

Altogether, these results, with all the limitations of a retrospective, single center based data set, provide initial support, for the first time in a clinical setting, for the simple sonographic monitoring of uncomplicated inactive CE4 and CE5 cysts of the liver that reach the inactive stage spontaneously. This particular distinction is important because the rate of reactivation of inactive cysts which became inactive as the result of therapy may instead be variable depending on the pre-treatment cyst stage, and the clinical management and follow-up of these “induced” inactive cysts is consequently different [20]. Importantly, our data highlight the need for a long-term follow-up of patients with inactive cysts, and for better patient education and patient recall system, to minimize the loss to follow-up and its possible consequences.

Our data confirm the marginal usefulness of serology in the follow-up of patients with CE cysts [15]. ELISA test was positive in 6 patients, and in 1 of these patients the test became positive over time without US evidence of reactivation, while the only case of reactivation had a persistently negative serology. These results were not due to the presence of cysts in extra-hepatic locations, as ruled out by abdominal and thoracic CT scans. This behavior is well known to clinicians caring for patients with CE, as serological tests may remain positive for years even after complete surgical removal, and do not imply the presence of active (re)infection [13], [21]. It is possible that temporary release of antigens from breaches in cyst integrity boosts antibody production in these cases. Acknowledging that serology has only a complementary role and cannot be used alone to guide the clinical management of CE has important consequences in terms of avoiding unnecessary treatment and reducing the patient's (and physician's) anxiety. Clinicians with little experience with this disease are often led to believe that positive serology automatically implies active disease even if cysts are nowhere to be found and this often translates into unnecessary and long-term administration of albendazole, with attendant side effects and cost.

To the best of our knowledge, this is the first time that long-term (median 5 years over a 19 years period) monitoring of inactive cysts of patients on a clinical rather than epidemiological basis has been reported, contributing to the debate on the best treatment for CE using a stage-specific approach. Moreover, our results shed further light on the natural history of CE and the relationship between cyst stage and antibody responses in a large cohort of patients where cysts reached the inactive stage spontaneously. Although large prospective and multi-centric studies will be needed to provide definitive recommendations for the clinical management of this patient category, our data from a relatively small series of well-defined patients with spontaneously inactivated asymptomatic cysts of the liver support the WHO-IWGE indications for these cases. These patients can be managed expectantly in the majority of cases, provided they can be monitored by regular US follow-up, without administering unnecessary drugs and incurring avoidable costs.
